# A randomized controlled trial of an exercise intervention targeting cardiovascular and metabolic risk factors for prostate cancer patients from the RADAR trial

**DOI:** 10.1186/1471-2407-9-419

**Published:** 2009-12-02

**Authors:** Daniel A Galvão, Nigel Spry, Dennis R Taaffe, James Denham, David Joseph, David S Lamb, Greg Levin, Gillian Duchesne, Robert U Newton

**Affiliations:** 1 Vario Health Institute, School of Exercise, Biomedical and Health Sciences, Edith Cowan University, Joondalup, WA, Australia; 2 Department of Radiation Oncology, Sir Charles Gairdner Hospital, Nedlands, WA, Australia; 3 Faculty of Medicine, University of Western Australia, Nedlands, WA, Australia; 4 The University of Queensland, School of Human Movement Studies, Brisbane, QLD, Australia; 5 School of Medicine and Public Health, University of Newcastle, Australia; 6 Newcastle Mater Hospital, Newcastle, Australia; 7 University of Otago, Wellington, New Zealand; 8 Peter MacCallum Cancer Centre, Melbourne, Australia

## Abstract

**Background:**

Androgen deprivation therapy leads to a number of adverse effects including deterioration of the musculoskeletal system and increased risk factors for cardiovascular and metabolic complications. The purpose of this study is to determine the effects, efficacy, retention and compliance of a physical exercise intervention in a large established cohort of prostate cancer patients from the Randomised Androgen Deprivation and Radiotherapy (RADAR) study. Specifically, we aim to compare short- and long-term effects of a prostate cancer-specific supervised exercise program to a standard public health physical activity strategy utilizing printed resources on cardiovascular and metabolic risk factors. Our primary outcomes are cardiorespiratory capacity, abdominal obesity, and lipid and glycemic control, while secondary outcomes include self-reported physical activity, quality of life and psychological distress.

**Methods/Design:**

Multi-site randomized controlled trial of 370 men from the RADAR study cohort undergoing treatment or previously treated for prostate cancer involving androgen deprivation therapy in the cities of Perth and Newcastle (Australia), and Wellington (New Zealand). Participants will be randomized to (1) supervised resistance/aerobic exercise or (2) printed material comprising general physical activity recommendations. Participants will then undergo progressive training for 6 months. Measurements for primary and secondary endpoints will take place at baseline, 6 months (end of intervention), and at 6 months follow-up.

**Discussion:**

This study uses a large existent cohort of patients and will generate valuable information as to the continuing effects of exercise specifically targeting cardiovascular function and disease risk, insulin metabolism, abdominal obesity, physical function, quality of life and psychological distress. We expect dissemination of the knowledge gained from this project to reduce risk factors for the development of co-morbid diseases commonly associated with androgen deprivation therapy such as cardiovascular disease, obesity, metabolic disease and diabetes, as well as improvements in physical and functional ability, and quality of life.

**Trial Registration:**

ACTRN12609000729224

## Background

Advancing age increases the vulnerability to cancer and the risk for other comorbid conditions (e.g. cardiovascular disease, diabetes, osteoporosis, arthritis and sarcopenia) [1] that can compromise physical function and independent living, ultimately culminating in death. The high prevalence of cancer and comorbidity-related conditions, apart from exacting a high personal, family and community cost, places a heavy burden on the health care system. Lifestyle interventions that can ameliorate toxicities of treatment, and improve ability to self care are seen as highly desirable.

Androgen deprivation therapy (ADT) leads to a number of adverse effects including deterioration of the musculoskeletal system and increased risk factors for cardiovascular and metabolic complications (e.g. negative lipoprotein profile, reduced insulin sensitivity, abdominal obesity) [2-7]. Moreover, investigators from four large recent observational studies, including our own current Australian cohort, found an increased incidence of myocardial infarction and metabolic complications following ADT [8-12]. This is of considerable concern to patients and clinicians as the co-morbidities exacerbated by ADT become a greater threat to survival than the prostate cancer itself.

Regular exercise is established for primary and secondary prevention of several chronic diseases including cardiovascular disease (CVD) and diabetes, and even premature death [13,14]. The protective effect of physical activity appears to be greater in individuals at highest risk for type 2 diabetes [15]. Notably, in regards to the risk for premature death, cardiorespiratory reserve appears to be more important than other well known CVD risk factors [16]. Men with prostate cancer undergoing ADT are recognized to gain fat mass, lose lean mass, and be subject to a series of adverse effects from therapy [5,7,9,17]. Hence these men could benefit from exercise by reducing risk factors for metabolic complications and therapy-related co-morbidities [9].

We have previously summarized all exercise interventions undertaken during and after cancer treatment [18]. Most of the experimental studies comprised patients with breast cancer and a mixture of other types of cancer, but did not include men with prostate cancer undertaking ADT [18]. Moreover, most exercise interventions included very low subject numbers, limited study designs and short-term intervention periods with modest or no follow-up periods. Recently, we [19,20] and others [21,22] in tightly controlled laboratory-based trials have demonstrated that exercise can prevent and even reverse adverse effects of androgen suppression. We have also recently reported specific exercise guidelines for prostate cancer patients that could potentially be incorporated in extensive population cohorts and community programs benefiting large numbers of patients [23]. However, to date there has been no large representative cohort study with prostate cancer patients undertaking exercise and this should be viewed as an immediate priority given the potential to reduce treatment toxicity, incidence of co-morbidity, mortality and substantially improve quality of life and well being. This study is unique as it applies findings from recent clinical trials [19-22] to a very large representative cohort of patients, the Randomised Androgen Deprivation and Radiotherapy (RADAR) study cohort. The RADAR study, examines the effect of adjuvant ADT on relapse free survival as well as other outcome measures in over 1000 men with prostate cancer at several centers in Australia and New Zealand. It will also allow us the opportunity to explore biological questions that would normally be too expensive to investigate (using data already collected by the RADAR study). The beneficial outcomes of the exercise intervention interaction, if substantiated, have the potential to alter clinical practice worldwide.

The purpose of this study is to determine the effects, efficacy, retention and compliance of a physical exercise intervention in a large established cohort of prostate cancer patients from the RADAR study. Specifically, we aim to compare short- and long-term effects of a prostate cancer-specific supervised exercise program to educational material of physical activity utilizing printed resources on cardiovascular and metabolic risk factors. Our primary outcomes are cardiorespiratory capacity, abdominal obesity, and lipid and glycemic control, while secondary outcomes include self-reported physical activity, quality of life and psychological distress. Our hypothesis is that compared to a standard physical activity recommendation (e.g. perform 30 minutes of moderate/vigorous physical activity 5 days of the week), the prostate cancer-specific supervised exercise intervention will elicit superior adaptations in: improving physical/cardiorespiratory capacity; body composition (reducing abdominal fat mass); glycemic control and lipid metabolism; enhancing self-reported physical activity, quality of life and psychological distress. This project has the potential to elicit positive health benefits for large numbers of men with prostate cancer. It is a lifestyle intervention that allows patients some control and personal responsibility and, as such, could easily be expanded to all such patients resulting in a considerable reduction in morbidity and mortality.

## Methods/Design

**Figure 1:**
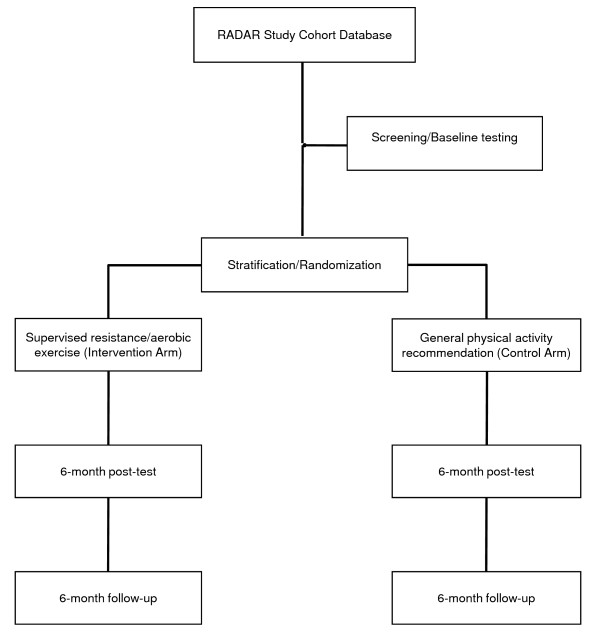
**CONSORT diagram**.

This is a randomized controlled trial in which all subjects will receive printed material which describes the importance of exercise for prostate cancer patients and outlines basic recommendations to increase their levels of physical activity (e.g. perform 30 minutes of moderate/vigorous physical activity 5 days of the week). However, only half of the subjects will be given access to regular sessions with an exercise physiologist and access to dedicated Internet resources over a period of 6 months (group 1). In the second 6-month period, group 1 will further have access to a booklet with detailed information about a home exercise program but will not receive exercise supervision (Figure 1). The group only receiving printed material about basic recommendations to increase levels of physical activity (control condition, group 2) will remain unchanged during the 6-month follow-up. This design is based on our experience over numerous exercise trials and has several advantages over a "no intervention" control group. First, in exercise research, subjects allocated to a true control group will either comply, drop out early, or take up exercise regardless of the instructions to maintain their usual lifestyle. The result is very low retention or worse, a severely contaminated dataset. At the completion of the study, group 2 participants (control condition) will be given access to all printed and Internet-based resources (including the booklet with detailed information about a home exercise prescription) developed during the course of the study to encourage exercise adoption.

### Subjects

370 men from the RADAR study cohort undergoing treatment or previously treated for prostate cancer involving ADT with no regular exercise (undertaking structured aerobic or resistance training less than two times per week) within the past 6 months will be recruited (Figure 1). Participants will have been treated or are currently being treated with ADT, able to walk 400-m and will require medical clearance from their physician (general practitioner). Exclusion criteria will include bone metastases, acute illness or any musculoskeletal, cardiovascular or neurological disorder that could inhibit or put them at risk from exercising. Participants with established but controlled CVD or type II diabetes will not be excluded from participation given the large benefits of exercise. Subjects will be recruited from the RADAR study database via a letter of invitation from their specialist and will then be contacted by an exercise physiologist accredited by the Australian Association for Exercise and Sport Science (AAESS) or Sport & Exercise Science New Zealand (SESNZ) in their respective geographical areas. Those entering the study will complete a health history questionnaire and undertake baseline measures prior to being randomized to either the supervised exercise group or the standard physical activity recommendation group. The protocol has been approved (ID: 3636-GALVÃO) by the University Human Research Ethics Committee and all subjects will provide written informed consent.

### Randomization and Stratification

**Figure 2:**
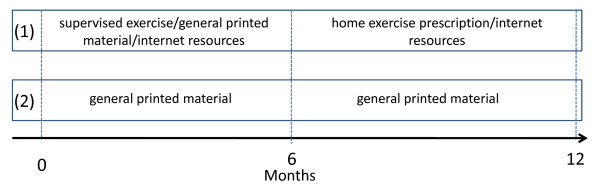
**Exercise interventions and timeline**.

Patients will be randomly allocated to the two treatment arms: (1) exercise and (2) standard physical activity recommendation in a ratio of 1:1, subject to maintaining approximate balance regarding stratification for age (<69, 69-74, >74), original RADAR study arm [(A) 6-month ADT and radiation; (B) 6-month ADT, radiation and 18-month bisphosphonate; (C) 18-month ADT and radiation; and (D) 18-month ADT, radiation and 18-month bisphosphonate], current levels of testosterone (<3, 3-8, >8), and waist circumference (< or > than 102 cm) (Figure 2).

### Exercise Intervention (group 1)

The exercise program will be undertaken twice weekly in small groups of participants and will be supervised by the exercise physiologist over 6 months. This group will also have access to Internet resources where they will be able to input information regarding their weekly physical activity. Sessions will comprise aerobic and resistance exercise using a variety of equipment. In addition, 2 exercise sessions will be completed at home each week comprising aerobic exercise (e.g. walking). The clinic sessions, which will commence with a warm-up and finish with a cool-down (comprising exercise on a cycle ergometer or treadmill at a relatively low intensity and stretching activities), will take approximately 60 minutes and will be conducted in local exercise facilities. The moderate- to high-intensity progressive resistance training regime will include 7 exercises that target the major upper and lower body muscle groups, which we have used previously in a number of studies including those with prostate cancer patients [19,24-27]. The exercises are the chest press, seated row, shoulder press, leg press, leg extension, leg curl and abdominal crunches. The resistance exercise program is designed to progress from 12 to 6-repetition maximum (RM) for 2 to 4 sets per exercise. Each clinic session will include 20-30 minutes of aerobic exercise using various modes such as walking or jogging on a treadmill, cycling or rowing stationary ergometers. Target intensity will be 70-85% of estimated maximum heart rate with individual heart rate monitors provided for each participant. During the 6-month follow-up period, this group will have access to a booklet with detailed information about a home exercise prescription but will no longer receive exercise supervision. The home exercise program combines resistance training (using body weight, rubber bands, and objects around the house as a resistance and is designed to replicate the exercises performed in the supervised sessions), aerobic training, and flexibility exercises.

### Standard Physical Activity Recommendation (group 2)

The standard physical activity recommendation group (control condition) will be instructed via printed material to perform the general recommendation of 30 minutes of moderate/vigorous physical activity 5 days per week (150 minutes per week of moderate activity) during the initial 6-month period as well as the 6-month follow-up period. At the completion of the study, this group will be given access to the booklet with detailed information about a home exercise prescription and internet resources developed during the course of the study to encourage exercise adoption. Instruction on performing the home-based exercises will be provided by the exercise physiologist (this will take place at the end of the 6-month follow-up).

### Study Endpoints

Measurements will take place at baseline, 6 months (end of the supervised exercise intervention period), and at 6 months follow-up. The assessment at 6 months follow-up is to determine the long-term effects and whether differences observed directly following the intervention are maintained in the exercise group when the booklet of standard physical activity recommendations is supplemented with the home exercise prescription.

### Aerobic Walking Capacity

As a measure of quantitative aerobic walking endurance capacity, subjects will undertake a timed 400-m walk, 10 laps out and back over a 20-m corridor path [19,28,29].

### Anthropometric Measures/Abdominal Obesity

Central adiposity will be assessed by waist circumference (WC) and hip circumference (HC) will also be determined [30]. WC will be measured at the level of the narrowest point between the lower costal (rib) border and the iliac crest. HC will be measured at the level of the greatest posterior protuberance of the buttocks which usually corresponds anteriorly to the level of the symphysis pubis. The waist-to-hip ratio will be determined and has been shown to be a strong indicator of atherosclerosis and risk of myocardial infarction [31,32]. Body mass index (kg/m^2^) will also be used to assess weight (kg) relative to height (m) and is a good indicator of obesity and metabolic and health-related problems.

### Blood Markers

Testosterone, prostate specific antigen (PSA), insulin, lipid profile, glucose and HbA_1c _levels will be measured commercially by accredited Australian National Association of Testing Authorities (NATA) and International Accreditation New Zealand (IANZ) laboratories.

### Self-reported Physical Activity

Self-reported physical activity will be assessed by the leisure score index from the Godin Leisure-Time Exercise Questionnaire. Overall physical function will also be assessed qualitatively using the SF-36 physical function sub-scale. In addition, participants will wear a pedometer for seven days at the three measurements time points to obtain a quantitative measure of steps per day.

### Quality of Life, Falls Self-Efficacy and Psychological Distress

Health-related quality of life will be measured using the EORTC QLQ-C30 and EORTC QLQ-PR25 as well as a health history questionnaire [3,33]. This validated instrument is an integrated system to assess quality of life in cancer patients and has been extensively employed in clinical trials [34]. The Brief Symptom Inventory-18 (BSI-18) will be used to assess psychological distress (Anxiety, Depression and Somatisation) [35]. Falls self-efficacy was determined using the activities-specific balance (ABC) confidence scale [36].

### Nutrition

Participants will be encouraged to maintain customary activity and dietary patterns and the mini nutritional assessment (MNA) instrument will be used to monitor nutritional status [37].

### Lower Body Physical Function

Lower body performance will be assessed by the repeated chair rise test, where subjects rise to a standing position five times as fast as possible without using their hands for support [19,25].

### Additional Measures for the Perth Site

In addition to the above measures, participants at the Perth site will have the following assessments undertaken at the same 3 time points.

### Body Composition

Whole body lean mass (LM), fat mass (FM), and percent fat will be assessed by dual-energy X-ray absorptiometry (DXA, Hologic Discovery A, Waltham, MA). From the whole body scan, upper limb, lower limb, and trunk LM and FM will be derived by manipulating segmental lines according to anatomical landmarks [38]. Upper limb LM and lower limb LM will then summed to derive appendicular skeletal muscle [39].

### Muscle Strength

Dynamic concentric muscle strength will be measured using the one repetition maximum (1-RM) method. The 1-RM is the maximal weight an individual can move through a full range of motion without change in body position other than that dictated by the specific exercise motion [25].

### Balance and Risk of Falling

A Neurocom Smart Balancemaster (Neurocom, OR, USA) will be used to assess static and dynamic balance. This device measures ground reaction force to track whole body centre of pressure and a tilting visual field and support platform to separate the visual, somatosensory and vestibular balance sense of the patient. As a measure of dynamic balance, subjects will also walk backwards 6-m and time taken will be assessed using electronic timing gates [25].

### Calculation of Sample Size and Statistical Analysis

The sample size estimate was based on projected changes in aerobic walking capacity (cardiorespiratory fitness) as measured by the 400-m walk as this study endpoint has been shown to be a strong predictor of mortality, CVD and mobility limitations in older adults [28,40,41]. To achieve 80% power at α = 0.05 (two-tailed), 142 subjects per group are required to demonstrate a 0.33 SD difference between groups at the end of the 6-month intervention and follow-up periods. Previous experience with exercise trials indicates an attrition rate of up to 30% over the course of the study period. Therefore, to ensure that we have sufficient subject numbers at the end of the intervention and follow-up periods, 185 subjects will be randomized to each of the groups (N = 370). Regarding the other outcomes, a sample size of 370 will account for expected differences. Data will be analysed using the SPSS statistical software package. Analyses will include standard descriptive statistics, Student's t tests, correlation and regression, and two-way (group × time) repeated measures ANOVA to examine differences between groups over time. All tests will be two-tailed and an alpha level of 0.05 will be applied as the criterion for statistical significance.

## Discussion

This will be the first exercise intervention undertaken in a large cohort of prostate cancer patients and will produce the strongest efficacy information to date. As this project uses a very large and representative cohort of patients and is longer than previous investigations in the area of exercise and cancer, we will gain valuable information as to the continuing effects of exercise specifically targeting cardiovascular and muscle function and disease risk, insulin metabolism, abdominal obesity, physical function, quality of life and mental health. Specifically we will be able to: 1) compare a large-scale supervised program of exercise to standard physical activity recommendations, and 2) examine the effect that a non-supervised, exercise-specific home-based program has on maintaining gains derived from supervised training. Importantly, this simple and cost effective intervention strategy may provide similar benefits to pharmaceutical interventions without exposing patients to additional potential side effects and cost. In terms of advancement of prostate cancer care, we expect dissemination of the knowledge gained from this project to reduce risk factors for the development of co-morbid diseases such as CVD, obesity, metabolic disease and diabetes, as well as improve physical and functional ability, and quality of life. In addition, such positive effects could significantly reduce health care costs. Lastly, by incorporating an extensively studied patient cohort, the RADAR cohort, other subsequent biological questions may be possible to address.

## Competing interests

The authors declare that they have no competing interests.

## Authors' contributions

DAG, NS, DRT, JD and RUN developed the study concept and protocols and initiated the project. DJ, DSL, and GL assisted in further development of the protocol. DAG, NS, DRT and RUN drafted the manuscript. NS, JD, DJ, DSL and GD will provide access to patients. DAG, RUN, NS, DRT, GL and RUN will implement the protocol and oversee collection of the data. All authors contributed and approved the final manuscript.

## Pre-publication history

The pre-publication history for this paper can be accessed here:

http://www.biomedcentral.com/1471-2407/9/419/prepub
